# Stable Expression and Characterization of an Optimized Mannose Receptor

**DOI:** 10.4172/2155-9899.1000330

**Published:** 2015-06-06

**Authors:** David J Vigerust, Sherell Vick, Virginia L Shepherd

**Affiliations:** 1 Department of Veterans Affairs Medical Center, USA; 2 Department of Pathology, Microbiology and Immunology, Vanderbilt University School of Medicine, Nashville TN 37212, USA

**Keywords:** Macrophages, Mannose receptor

## Abstract

The mannose receptor (MR) is a macrophage surface receptor that recognizes pathogen associated molecular patterns (PAMPs) from a diverse array of bacterial, fungal and viral pathogens. Functional studies of the MR are hampered by the scarcity of human cell lines that express the receptor. Current model systems available for the study of MR biology often demonstrate low levels of expression and do not retain many of the classical MR properties. Although several laboratories have reported transient and stable expression of MR from plasmids, preliminary data from our laboratory suggests that these plasmids produce a protein that lacks critical domains and is often not stable over time. In this current report we describe the generation and characterization of a novel human codon-optimized system for transient and stable MR expression. Rare codons and sequences that contribute to mRNA instability were modified to produce mRNA that is qualitatively and quantitatively improved. Confocal imaging of the transient and stably expressed optimized receptor demonstrates a distribution consistent with previous reports. To demonstrate the functional characteristics of the optimized receptor, we further show that the introduction of codon-optimized MR plasmid can confer MR-associated phagocytosis of *S. aureus* to non-phagocytic HeLa cells. We show that three molecules participate in the engagement and internalization of *S. aureus*. MR was found to colocalize with Toll-like receptor 2 (TLR2) and Rab5 following exposure to pHrodo-stained *S. aureus*, suggesting cooperation among the three molecules to engage and internalize the bacterial particle. This study describes a transfection capable, optimized MR receptor with functional characteristics similar to the wild type receptor and further demonstrates a new system for the continued study of MR biology and function.

## Introduction

The mannose receptor (MR) is a 175 kDa type I transmembrane protein and the prototype member of the C-type lectin family of proteins that includes the MR, Endo180, DEC205 and the phospholipase A2 receptor. The MR was initially described by Stahl and coworkers as a cell surface receptor on macrophages that is involved in the clearance of extracellular hydrolases [1], capture and clearance of pathogens [2], capture of foreign antigens for presentation to MHC-II compartments [3,4], clearance of glycoprotein hormones [5], clearance of extracellular peroxidases [6,7], endocytosis of lysosomal acid phosphatase [8], and regulation of glycoprotein homeostasis [9]. Structurally, the receptor contains a cysteine-rich domain, fibronectin type II repeat, and eight carbohydrate recognition domains (CRD), of which 4, 5 and 7 are reported to be invaluable to the binding and internalization of ligands with exposed oligosaccharides terminating in mannose, fucose or N-acetylglucosamine [10].

A defining feature of the MR is the capacity for rapid internalization from the plasma membrane via a clathrin-mediated mechanism that delivers the receptors and engaged cargo to the endocytic pathway [11,12]. Studies have shown that the MR engages ligands and mediates their internalization via receptor-mediated endocytosis [13,14], and participates in phagocytosis of mannosylated pathogens [15,16]. Ligands containing terminal sugars such as mannose bind at neutral pH to the MR at the cell surface and are brought into the cell. After dissociation in the acidic endosomal environment, ligands are delivered to the lysosomal compartment for degradation and the MR returns to the cell surface [17]. Reports suggest that 10–30% of the receptor at steady state resides on the cell surface and the remaining 70–90% is located in intracellular pools. The MR has a long half-life (>30 hours), and makes ten or more rounds of ligand internalization each hour [18].

In addition to their endocytic properties, several members of the MR family of molecules participate in phagocytosis, a function vital to the role of the macrophage in the innate immune response. Macrophages are present in virtually all tissues and are especially prominent in mucosal tissues. Within the context of innate immunity, macrophages are among the first cells to encounter an invading microorganism. The recognition capacity of the MR is broad and allows for the binding and uptake of a diverse array of pathogens including *Schistosoma mansoni* [19], *Paracoccidioides brasiliensis* [20,21] *Francisella tularensis* [22] *Candida albicans* [23], *Leishmania donovani* [24,25], *Mycobacterium tuberculosis* [26–28], *Pneumocystis jirovecii* [29], *Klebsiella pneumoniae* [30], HIV [31–33], Dengue virus [34], Hepatitis B [35], and influenza A [36,37]. Although mannose is not a common terminal carbohydrate residue on the cell surface of mammalian-derived proteins, it is commonly found on the surface of a variety of pathogens, thus allowing the MR to distinguish self from non-self through carbohydrate recognition.

The MR and MARCO (macrophage receptor with collagenous structure) have been identified as an opsonin-independent phagocytic receptor in the lung for *S. pneumonia* [38,39]. Dorrington et al. reported that MARCO is required for TLR2- mediated responses to *S. pneumoniae* [40] just as MR was reported to mediate internalization of *S. pneumoniae* in Schwann cells [41]. Additionally, recent investigation reveals that concentrations of soluble MR are much higher in patients with pneumococcal bacteremia suggesting macrophage activation and placing MR as a new biomarker for invasive disease [42]. Mannose receptor and TLR2 are likewise involved in the recognition and response to *Paracoccidioides brasiliensis* [20,21]. During the course of particle internalization Rab5a and MR have been shown to cooperate. Mannose receptor levels and MR-mediated endocytosis are enhanced significantly in the presence of Rab5a [43]. Both endocytic and phagocytic events in the macrophage are activated by Rab5 [44]. The association of these molecules under endocytic and phagocytic conditions led to the hypothesis that these molecules would cooperate in bacterial clearance.

Few commonly used continuous macrophage cell lines express a functional MR, hampering studies on MR structure and function. Human THP-1 monocyte cells have been reported to express the MR following stimulation with phorbol myristate acetate (PMA) or phorbol ester 12-O-tetradecanoylphorbol-13-acetate (TPA) [45,46]. Diment et al. reported that MR expression could be induced on murine J774 cells following treatment with azacytidine, and our laboratory has described the characterization of the MR on the rat NR8383 macrophage line and the FDRC dendritic cell line [47,48]. Although these cell lines have provided model systems for MR studies, expression is often low and not all properties of the receptor are retained in these altered cells. Further complicating functional MR studies is the lack of an expression system that has been shown to result in production of a functional MR. Although several studies have reported transient and stable expression, preliminary work from our laboratory suggests that the MR protein that is produced lacks certain domains in the cytoplasmic tail, preventing accurate studies of trafficking and signaling mediated by the MR. A primary focus in our laboratory has been the study of the domains in the MR that mediate specific functions such as endocytosis and phagocytosis. To continue these studies, we have generated a codon-optimized MR cDNA that, when expressed, produces a full-length and functional MR. In the current report we describe the characterization of this codon-optimized MR cDNA and its stable expression in a continuous human cell line.

## Materials and Methods

### Codon optimization of the mannose receptor

Previous studies in our laboratory indicated that the wild type MR was difficult to express in mammalian cells and that the plasmid was unstable during long-term storage. Based on work from a number of laboratories indicating that specific sequences in mammalian genes contribute to difficulty in subsequent expression [49–51], we examined the MR sequence to determine codon bias, using the method of codon adaptation measurement described by Sharp and Li [52]. To identify single rare codons and rare codon clusters, online databases such as the Rare Codon Calculator (RaCC) were used. The MR gene optimized for mammalian codon usage with flanking *Bam*HI and EcoRI sites was synthesized by GeneArt (Regensburg, Germany) and cloned into a pcDNA 3.0 vector.

### Plasmids and antibodies

Anti-MR monoclonal antibody was obtained from BD Biosciences (San Jose CA), and anti-toll-like receptor (TLR)-2 and TLR-4 antibodies were obtained from Santa Cruz Biotechnology (Santa Cruz, CA). Rab5 antibody was obtained from Cell Signaling Technology (Danvers, MA). Secondary antibodies conjugated to Alexa dyes 488, 555, and 647 for use in imaging studies were obtained from Invitrogen (Carlsbad CA). Phycoerythrin (PE)- or fluorescein isothyocyanate (FITC)-conjugated antibodies for use in fluorescent-activated cell sorting (FACS) were obtained from BD Biosciences (San Jose CA).

### Transfection of cells

Cells were transfected with FuGENE HD (Roche) transfection reagent according to the manufacturer’s directions. Briefly, cells were cultured at a concentration of 2 × 10^6^ cells per P-100 dish overnight. Plasmid DNA (10 µg) was incubated with transfection reagent and added to cells. The cells were incubated overnight at 5% CO_2_ and 37° C. Following incubation, the cells were washed and DMEM (Gibco, Carlsbad CA) with 10% FBS and antibiotics was added. Cells were collected at 24 and 48 hours post-transfection for flow cytometry, imaging or immunoblot analysis. Cells were cultured in the presence of 1 mg/ml of G418 sulfate (Mediatech, Manassas VA) for selection of stable transfectants.

### Flow cytometry and confocal microscopy

Flow cytometry was performed using a FACSCaliber (BD Biosciences, San Jose CA) bench top analyzer in the Tennessee Valley Health System, Department of Veteran Affairs cytometry core facility. Cells were stained with FITC- or PE-conjugated anti-MR polyclonal antibodies as follows: cells were collected by centrifugation, followed by suspension in staining buffer (1% BSA, 0.1% sodium azide, 0.5% normal goat serum in PBS). Normal goat serum was included in the stain buffer to saturate Fc receptors and minimize non-specific fluorescence. The appropriate concentrations of FITC- or PE-conjugated antibodies were diluted in staining buffer and added to the cells. An isotype-matched control was also performed to account for non-specific fluorescence, and an unstained sample was included to account for auto fluorescence. The cells were incubated for 20 minutes in the dark at 4° C, and then washed twice with staining buffer followed by fixation in 500 µl of 2% buffered paraformalin. The modulation of cell surface receptor density was represented as the percent change in mean fluorescence intensity as compared to the control. The number of events acquired for each sample was 3×10^4^ and cells were analyzed on a Becton Dickinson FACScan flow cytometer using CellQuest software or WinMDI FACS analysis software from J. Trotter (Scripps Research Institute, La Jolla CA).

Confocal microscopy was performed using a Zeiss LSM-510 confocal laser scanning inverted microscope in the Vanderbilt University imaging core facility. Cells were plated at a concentration of 2 × 10^5^ cells per 35 mm glass bottom Mattek tissue culture dish. Following treatment or incubation, cells were fixed in 2% buffered paraformalin followed by permeabilization with ice-cold methanol. Cells were then stained in a two-step process using mouse monoclonal anti-MR antibody followed by either goat anti-mouse Alexa-488 or Alexa-555 (Invitrogen, Carlsbad CA). The nucleus was stained for visualization with ToPro-3 (Invitrogen, Carlsbad CA). Cells were mounted in Aqua-polymount (Polysciences, Warrington PA) between coverslips. Images were collected at 520 nm using a 63× or 100× oil-immersion objective. Image processing was performed using Adobe Photoshop CS imaging software.

### Phagocytosis assay

Phagocytosis was assayed via uptake of pHrodo *S. aureus* BioParticles (Invitrogen, Eugene, OR). BioParticle conjugates are novel, no wash fluorogenic particles that are used for quantitative measurement of phagocytosis. Cells were cultured in complete media for 24 hours at a concentration of 2 × 10^5^ in Mattek tissue culture dishes. BioParticles prepared per manufacturer instructions were added to cells at a 5:1 ratio and cells plus particles incubated at 37°C for 30 minutes to allow for uptake. Cells were washed to remove unattached BioParticles, fixed in 2% paraformalin, stained with Alexa-488 conjugated anti-MR antibody, and prepared for confocal microscopy. Samples were examined by confocal microscopy using anti-MR Alexa-488, or Alexa 647-conjugated antibody against anti-TLR2 or Rab antibodies.

### Immunoblot analysis

Prior to transfection, cells were plated at a density of 5 × 10^5^ cells per well in 24-well plates or 1 × 10^6^ per P-100 tissue culture dish and incubated overnight in complete RPMI media. Following overnight incubation, fresh media was added and transfection or infection was performed. Following treatment, the cells were lysed in lysis buffer containing 0.5% NP-40. Total cellular protein was determined using the bicinchoninic acid (BCA) protein assay (Pierce, Rockford IL) and 10 µg of total protein was resolved by electrophoresis on a 7.5% SDS-PAGE gel at 150 volts constant at 4o C. Proteins were transferred to nitrocellulose at 100 volts followed by blocking in Licor Western blot block buffer. Immunoblot analysis was also performed on the Licor Odyssey platform using monoclonal anti-MR antibodies and goat anti-mouse IR dyes (Lincoln NE).

## Results

### Design of an MR cDNA for high-level expression in mammalian cells

**Figure 1:**
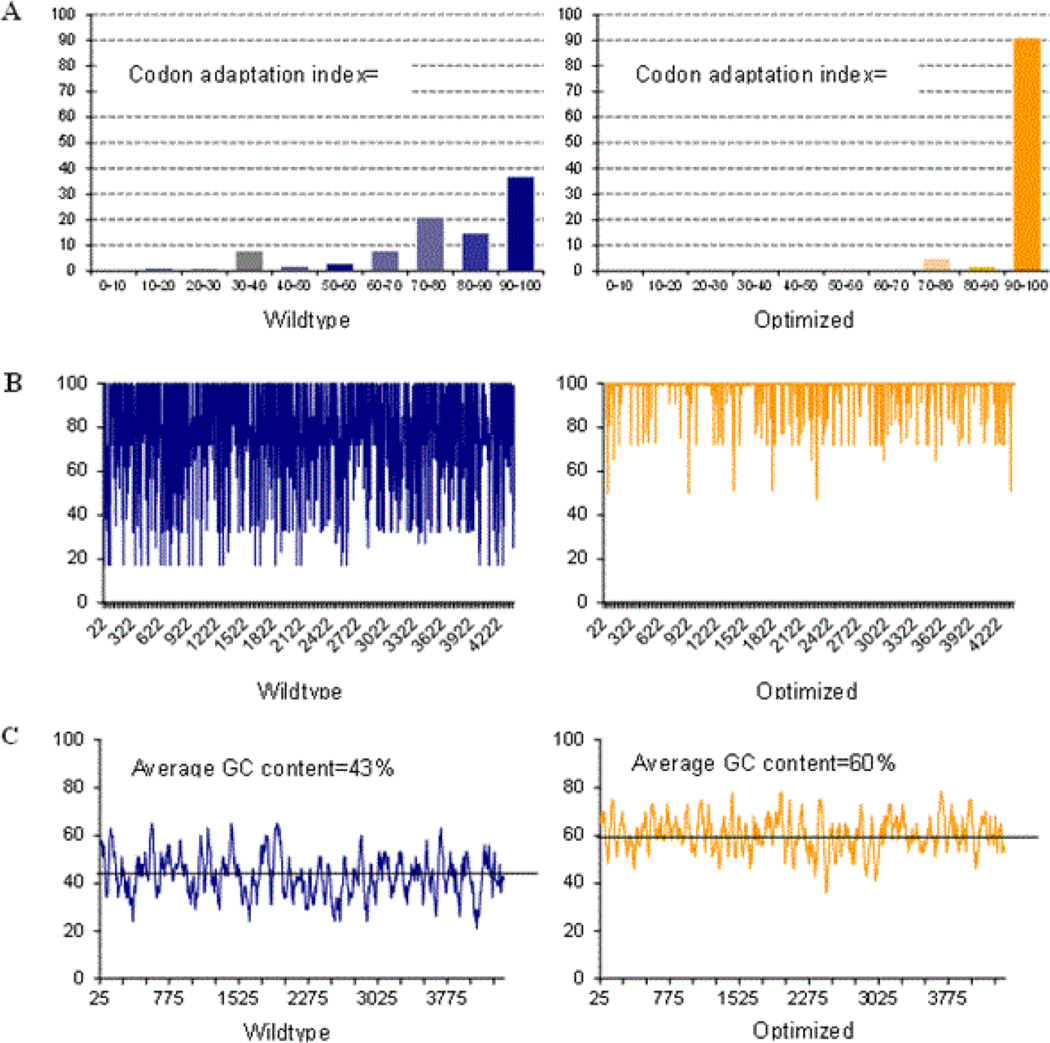
Codon optimization of human MR. (A). The histograms show the percentage of sequence codons, which fall into a certain quality class. The quality value of the most often used codon for a given amino acid in the desired expression system is set to 100, the remaining codons are scaled accordingly [52]. (B). The plots show the quality of the used codon at the indicated codon position. (C). The plots show the GC content in a 40 bp window centered at the indicated nucleotide position.

**Figure 2:**
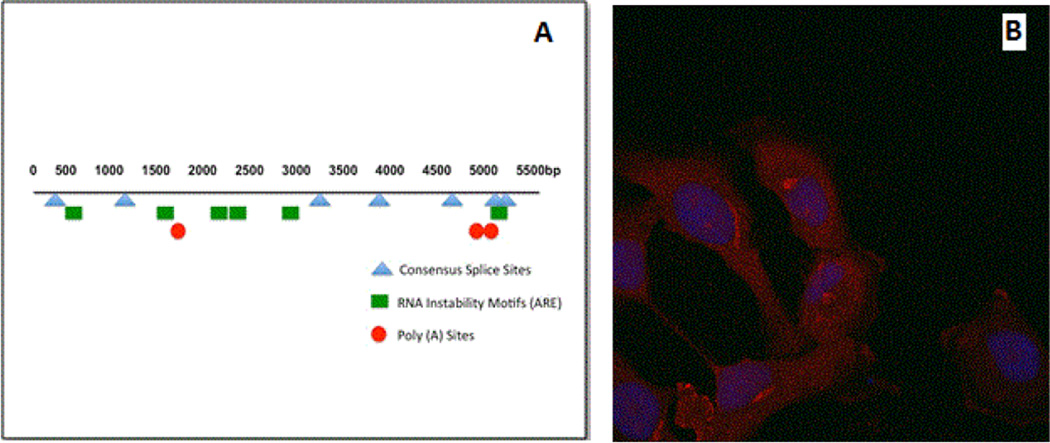
Negative cis-acting motifs and transient transfectants. (A). Schematic representation of the relative positions within the MR gene where negative cis-acting motifs were removed. (B). Transient transfection of HeLa and 293T cells was performed with gene optimized MR. Following incubation, cells were stained with monoclonal anti-MR and secondary stained with goat anti-mouse Alexa 555. Nuclear staining is achieved with ToPro-3. Images were captured with a 63× oil objective on the Zeiss LSM-510 (B) Demonstrates the distribution of MR in HeLa cells transfected with OptMR after 48 hours incubation.

Several recent studies have shown that rational design of mammalian genes can lead to higher level expression of heterologous genes [53]. For example, rare codons can dramatically limit mammalian protein expression in *E. coli*-based systems. The most commonly described codons include arginine (AGG, AGA, CGA), leucine (CTA), isoleucine (ATA), proline (CCC, and glycine (GGA). If these codons occur at a frequency higher than 1%, it is likely that translation will be negatively affected. In addition, clusters of these codons can reduce both the quantity and quality of synthesized protein [54,55]. The wild type human MR gene contains all of these codons, with significantly high levels of glycine and arginine rare codons, as well as doublet and triplet clusters as shown in Table 1. Data in Figure 1A shows the codon adaptation index (CAI) for the wild type and optimized MR sequence. In this measure, a value of 1 indicates the use of more common codons; any decrease in this number indicates more usage of rare codons. The CAI of 0.73 for the wild type MR correlates with the high percentage of rare codon usage as shown in Table 1, with the optimized MR sequence reaching almost 1. Data from a recent study suggested that wild type genes with a CAI of less than 0.78 were much less likely to properly express. As the CAI approached 1 through optimization, the genes demonstrated high levels of expression suggesting that the CAI was a strong measure of successful expression in mammalian cells [51]. The codon quality at each nucleotide position is shown in Figure 1B. In addition, other studies have reported that low GC content as found in the MR (43%; Figure 1C) can lead to rapid mRNA turnover. Moreover, several negative cis-acting motifs were found which have been reported to hamper expression in mammals (Table 2; Figure 2A) [49,56–58].

Based on these observations, and the known difficulty in achieving high levels of transient and stable expression from plasmids expressing the wild type MR sequence, the gene was submitted to GeneArt for optimization to enhance mammalian expression. Analysis of the resulting cDNA sequence demonstrated that negative cis-acting sites had been avoided, including internal TATA-boxes, chi-sites and ribosomal entry sites, AT-rich and GC-rich sequence stretches, repeat sequences and RNA secondary structures, (cryptic) splice donor and acceptor sites, and branch points which may negatively influence expression [49,50,56]. Additionally, the GC-content was increased to 60% to prolong mRNA half-life (Figure 1C). Codon usage was further adapted to the preferred for Homo sapiens, resulting in a high codon adaption index value. The optimized gene was modified to contain a Kozac sequence to increase translational initiation and two STOP codons to ensure efficient termination. The final product was shown to have a maximum nucleotide identity of 75% compared to the wild type sequence archived in Genbank. The sequence was cloned into the pcDNA 3.0 mammalian expression vector for transient and stable transfection into human cells and designated optMR for these studies.

### Transient expression of OptMR in 293T and HeLa cells

Transient expression of optMR in HeLa cells demonstrated that the receptor protein was widely distributed within the cell with focal accumulations in the perinuclear region consistent with ER and Golgi localization (Figure 2B). The expression of optMR was robust and reproducible and is consistent with patterns of staining that are seen with primary macrophages and with the human hybridoma cell line 43MR [59].

### Stable expression of OptMR in HeLa Cells

**Figures 3:**
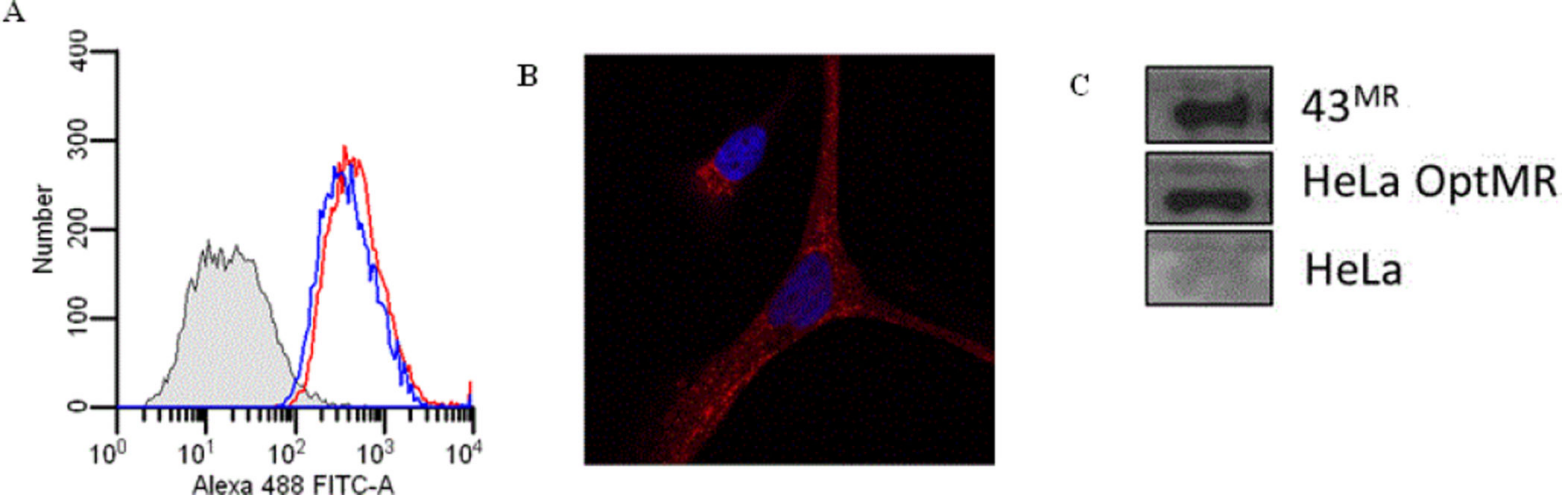
Stable transfection of HeLa cells was performed with gene optimized MR. Stables were selected with 1 mg/ml of G418 sulfate. The cells were incubated under selection pressure for several weeks with change of media and addition of G418 sulfate every other day. Following selection, cells were harvested and stained with monoclonal anti-MR and secondary stained with goat anti-mouse Alexa 488 or 555. Nuclear staining is achieved with ToPro-3. Images were captured with a 63× oil objective on the Zeiss LSM-510 (A) Demonstrates the cytometric profile of MR and TLR2 in HeLa cells stably expressing OptMR after in 1mg/ml G418. Shaded histogram represents the unstained control, TLR2 expression in blue histogram and MR expression in the red histogram. (B) Demonstrates the distribution of MR within stable transfectants. (C) Western blot analysis was performed on cell lysate from stable transfectants.

The optMR plasmid was transfected into HeLa cells and stable transfectants were selected using G418. Flow cytometry was used to determine the presence and the quantity of MR on the surface of stable cells (Figure 3A), and intracellular localization was analyzed by confocal microscopy. Work from previous studies has reported that the MR is expressed on the surface of macrophages, with a significant portion localized in intracellular compartments including endosomes, phagosomes, and multivesicular bodies [12,43]. As shown in Figure 3B, the stably expressed receptor is uniformly distributed throughout the cytoplasm with small aggregates (Figure 3B), similar to transient expression (Figure 2B) and previous reports in primary macrophages [60]. Western blot analysis of HeLa cells expressing optMR showed that the protein expressed is approximately 175 kDa (data not shown) and is recognized by anti-MR antibody and produced as strong a signal as the MR found in macrophages and in the MR expressing human hybridoma cell line 43MR (Figure 3C) [59].

### Engagement and phagocytic uptake of *Staphylococcus aureus*

**Figure 4:**
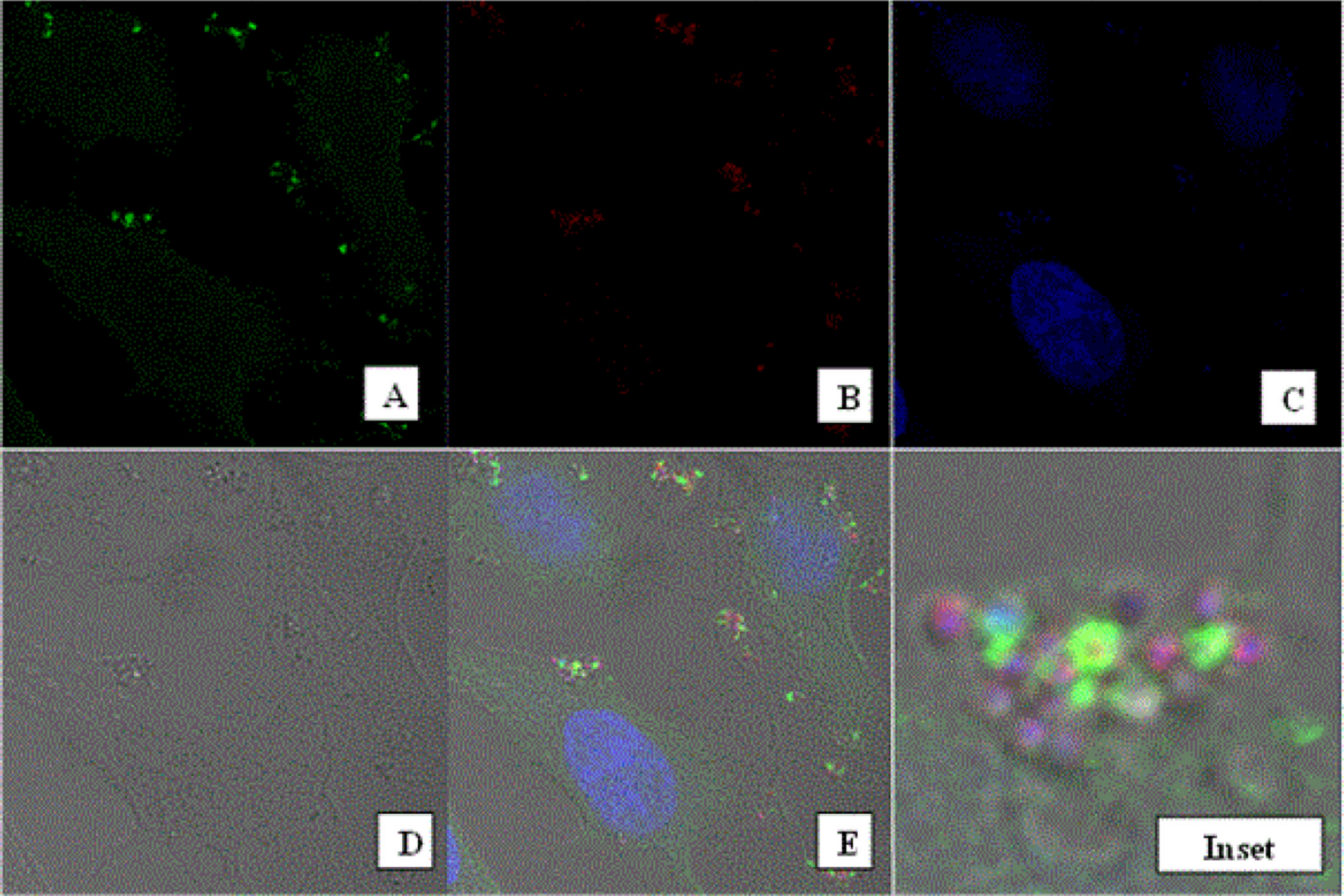
Phagocytosis of Staphylococcus aureus particles. HeLa OptMR stable transfectants were incubated with approximately 5:1 ratio of pHrodo SA particles per cell for 30 minutes. Following incubation, cells were stained with monoclonal anti-MR and secondary stained with goat anti-mouse Alexa 488. Images were captured with a 63× oil objective on the Zeiss LSM-510 (A). pHrodo SA particles are shown (B). Nuclear staining was achieved with ToPro-3 (C). Brightfield view of the cells (D). Merge panel (E). Panel F shows the digital zoom of the designated inset in panel E.

A key function of the MR is the internalization of microorganisms through interaction of the receptor with exposed mannose groups on the foreign particle surface. We therefore examined the ability of the expressed optMR to mediate phagocytosis of a particulate ligand. Stable transfectants were exposed to pHrodo-labeled *Staphylococcus aureus* (SA) for varying time periods ranging from ten minutes to several hours. Since the pHrodo-SA particle is sensitive to pH, only those particles that have been phagocytosed and localized to an intracellular acidic compartment will emit fluorescence. As shown in Figure 4E, as early as ten minutes post exposure, significant accumulations of the MR are seen at locations where the bacteria is located below the membrane. In several cases, the bacterial particle is encircled with MR in this particular focal plane. The brightfield image (Figure 4D) clearly shows that the particles are within the cell margins. These data suggest that the receptor is redistributed from a diffuse cytoplasmic localization to sites where challenge is present. Close analysis of the ring structure demonstrates that it is composed of several small accumulations or foci of MR. In many cases, the MR can clearly be seen to opsonize the particle.

### Receptor co-localization with Rab5 and TLR2

**Figure 5:**
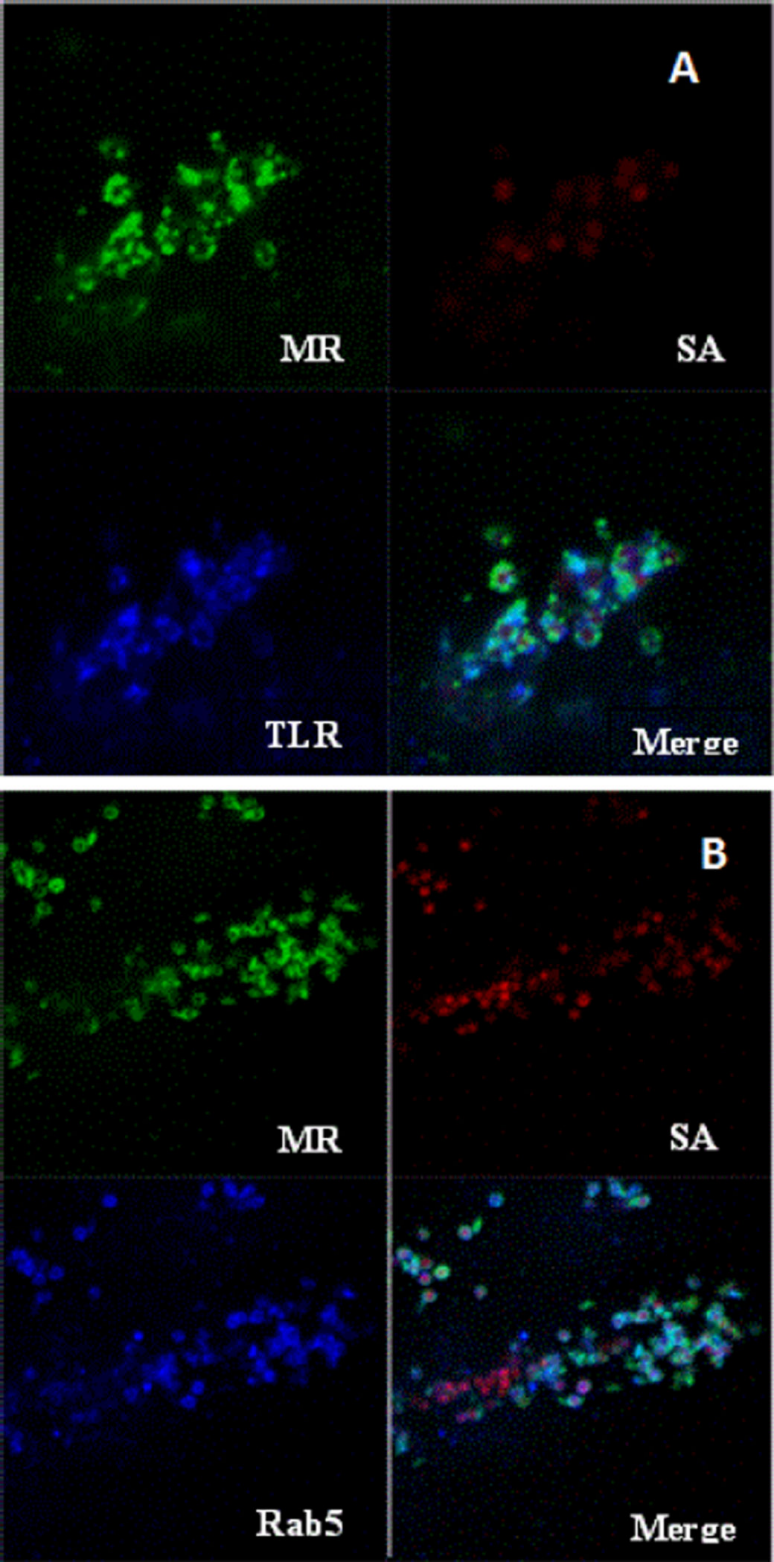
Co-Localization of MR with cellular proteins TLR2 and Rab5. HeLa OptMR stable transfectants were incubated with approximately five pHrodo SA particles per cell for 30 minutes. Following incubation, cells were stained with monoclonal anti-MR and rabbit polyclonal anti-TLR2 (A) or Rab5a (B). Secondary staining with goat anti-mouse Alexa 488 and goat anti-rabbit 647. Images were captured with a 100× oil objective on the Zeiss LSM-510.

Further analysis suggests that the MR is participating with other cellular proteins to engage and internalize the SA particle. *S. aureus* has been previously described to be engaged by TLR2 and so we proceeded to determine if TLR2 was expressed in the MR stable HeLa cell line. The results of flow cytometry demonstrate that the relative levels of TLR2 on the cell surface of our stable cell line is comparable to the levels of MR expressed on the cell surface (Figure 3A) Staining of SA exposed HeLa OptMR with antibodies for toll-like receptor (TLR) 2 and 4 demonstrated that TLR2 but not TLR4 colocalized with MR. Results from these co-localization experiments demonstrated that MR and TLR4 did not co-localize together (data not shown) but TLR2 was found to traffic to the particle and encircle the SA (Figure 5A) along with the MR. Co-localization experiments were also performed with the Rab proteins 5 (Figure 5B) and 7. Rab5 and MR both likewise appear to traffic to and encircle the bacterial particle resulting in co-localization of the MR with TLR2 and Rab5. Infection of SA exposed cells did not result in the localization of TLR4 to the particle nor did TLR4 colocalize with MR. Rab 7 was also tested and did not colocalize with the MR or the SA particle.

## Discussion

The macrophage mannose receptor has been shown by a number of different laboratories to be a key receptor in the clearance of extracellular hydrolases and peroxidases, and to facilitate entry of numerous pathogens. Studies on the mechanisms underlying these processes including intracellular trafficking and interaction with potential cellular cofactors has been hampered by the difficulty in our laboratory and others to express a functional receptor from the mouse or human cDNA sequence [61]. Indeed, preliminary work from our laboratory suggests that expression of the MR from currently available cDNA plasmids results in a protein that lacks some key structural sequences and/or functions. We examined the MR gene to determine if specific sequences might be preventing high-level expression of MR protein. In the current study, we have developed an MR cDNA for optimal expression in mammalian cells based on eliminating rare codons, increasing G/C content, and removing other RNA structural features that might limit expression. The result is a stable MR cDNA that can be expressed in *E. coli*, and can be transiently and stably expressed in human HeLa and 293T cells. The resulting MR protein expressed by HeLa and 293T cells has been structurally and functionally characterized, and appears to share many of the same features as the wild-type MR.

One of the first indications that the MR gene sequence itself might be hampering expression studies was the observation in our laboratory that the pcDNA3 vector itself was not stable upon storage, and could not be amplified in an *E. coli* -based system. In addition, any small amount of cDNA that could be isolated was not expressed to any measurable level in mammalian cells. Reports from other laboratories have suggested that selection of synonymous codons for a specific amino acid is not consistent among organisms, and may depend on a variety of factors including tRNA levels, G/C content, mutation frequency and patterns, and gene expression level [53]. Most often the codon usage presents problems when expression of heterologous genes is attempted, as in mammalian gene expression in E. coli or viral gene expression in mammalian cells. In our laboratory, we have previously encountered difficulties in expressing the MR cDNA in both E. coli systems as well as mammalian cells. Although little information is available concerning the effect of codon bias between different human cells and tissues, work from Plotkin and others suggest that codon usage could be tissue-specific [62]. One study of MR expression has been in the human macrophage cell line HL60. Results from this study suggest that control of cell surface expression of the MR may be tissue-and/or cell-specific [63].

In our current study we examined the expression of the MR using a cDNA optimized for high-level expression. The resulting cDNA, produced commercially by GeneArt, has lower rare codon usage, higher GC content, and reduced negative cis acting elements, all contributors to low protein expression (Tables 1 and 2; Figure 1). This approach has been used by a number of researchers to optimize expression of variety of plant and animal proteins, including a large study by Fath et al. where a direct comparison of 50 wild type and sequence optimized genes was performed [51]. High-level expression has resulted in enhanced ability to examine protein function in in vitro model systems. Optimization has also been applied clinically. In a recent study by Kosovac et al. sequence optimization was used to achieve high level and long-term expression of erythropoietin for enhanced gene delivery in vivo [64]. This report also showed high level expression correlated with higher GC content (CpG dinucleotides), consistent with the finding in the current study that increased GC content led to higher MR expression [64,65].

The wild type MR gene uses rare codons with a high frequency and contains a GC content that is quite low. These features affect expression by facilitating rapid mRNA turnover. Moreover, several negatively cis-acting motifs, which might hamper expression in mammals, were discovered. Following optimization of the gene, negative cis-acting sites (such as splice sites, poly (A) signals, etc.) were removed which may have previously negatively influenced protein expression (Table 1). GC-content was increased to prolong mRNA half-life (Figure 1C). Further, codon usage was adapted to the bias of *Homo sapiens* resulting in a high codon adaption index value of 0.97 as compared to the wild type MR gene which had a codon adaption index of 0.73 (Figures 1A and 1B). The parameter CAI (codon adaptation index) describes how well the codons match the codon usage preference of the target organism. Thus, a CAI of 1.0 would be perfect [66]. However, a CAI of>0.9 is considered as very good (i.e. allowing high expression). The optimized gene therefore allows for high expression rates in HeLa cells (Figure 2). We determined that in transient conditions the receptor expresses efficiently and with a pattern of cellular distribution consistent with previous characterization of MR expression. The optimized MR protein was shown to be of the same molecular weight and was strongly recognized by anti-MR antibodies. The Western blot analysis demonstrated a protein of the same physical size and reactivity as the MR found in primary macrophages and in a previously described human hybridoma cell line that also expresses functional MR [59].

To determine if the codon-optimized MR can confer a phagocytic capacity, we have employed a pathogen of global concern and interest to illustrate the phagocytic properties of transfected cells. Serious infections from *Staphylococcus aureus* strains are increasing in recent years in populations at risk [67]. *S. aureus* is a normal human commensal and carriage species that typically results in infections of the skin and soft tissues [68]. Less frequently infections result in more serious outcomes, including sepsis, necrotizing fasciitis, osteomyelitis, and pneumonia [69]. MR stably expressed in HeLa cells was used to demonstrate that three molecules participate in the initial encounter between *S. aureus* and the healthy cell. MR co-localized with both TLR2 and Rab5 following exposure to pHrodo-stained *S. aureus*, suggesting cooperation among the three molecules to engage and internalize the bacterial particle. The cooperation of the MR with TLRs has recently been described for a vaiety of human pathogens such as *Pseudomonas aeruginosa* [70], *Paracoccidioides brasiliensis* [20], and *Mycobacterium tuberculosis* [28]. Cooperation has also been demonstrated after exposure to non-pathogenic bacteria [71] where it was shown that Gram positive bacteria activated the TLR2 pathway and the Gram negative bacteria activated the TLR4 pathway. Consistent with these findings we found that cooperation between MR and TLR2 following exposure to *S. aureus* but no cooperation was seen with TLR4. The interaction of MR with TLRs may prove to be very important in cases where secondary bacterial infection following virus infection as has been described. Bacterial-viral synergism contributing to serious disease has been described in HIV, influenza, dengue and respiratory syncytial virus [72–75]. Interestingly, these viruses also employ interaction with the MR as part of their entry and as a consequence alter the expression of MR on the surface of macrophages. The alteration of MR expression on the cell surface may prevent the appropriate recognition and clearance of bacteria providing an avenue for colonization and serious infection. The development of a receptor optimized for expression in a variety of cell types provides an important tool to better understand the sequences involved in microbial recognition, signalling and trafficking. We have established here a construct that biochemically, structurally and most importantly behaves exactly like the wild type MR found in macrophages.

## Figures and Tables

**Table 1:** Rare Codon Usage in the Wild type Mannose Receptor

Amino Acid/Rare Codons	#	%
**Arginine: AGA, AGG,** **CGA**	44	3
**Glycine: GGA**	39	2.7
**Isoleucine: ATA**	13	0.9
**Proline: CCC**	12	0.8
**Leucine: CTA**	11	0.8
**Doublets**	4	0.2
**Triplets**	3	0.2

**Table 2:** Comparison of wild type and optimized MR gene motifs

	Wild type MR	Optimized MR
**Prokaryotic inhibitory motifs**	12	0
**Poly (A) sites**	3	0
**Consensus (cryptic) splice donor** **sites**	4 (6)	0
**RNA instability motifs (ARE)**	8	0
